# Supplementary material for: Gene co-expression network for analysis of plasma exosomal miRNAs in the elderly as markers of aging and cognitive decline
Source: PeerJ. 2020 Jan 6;8:e8318. doi: 10.7717/peerj.8318 (PMC6951281; doi:10.7717/peerj.8318)
Supplement: Supplemental Information 1 [file peerj-08-8318-s001.docx]

Supplement materials 1

The main principle of WGCNA algorithms

WGCNA is a systems biology method that uses gene expression data to construct a scale-free network. The basic idea is as follows. First, construct a gene expression similarity matrix, that is, calculate the absolute value of the Pearson correlation coefficient between the two genes, and use Formula 1 to calculate the Pearson correlation coefficient between gene i and gene j, where i and j are the ith The amount of expression of the gene and the jth gene.

Equation 1: S_ij_=|(1+cor(x_i_+y_j_))/2|
Then, using Equation 2, the gene expression similarity matrix is ​​converted into an adjacency matrix, and the network type is signed, where β is a soft threshold, which is actually the Pearson correlation coefficient β of each pair of genes. This step can strengthen strong correlation and weaken weak correlation from the index level.

Equation 2: α_ij_=|(1+cor(x_i_+y_j_))/2|^β^
The next step is to convert the adjacency matrix into a topological matrix using Equation 3. The topological overlap measure (TOM) is used to describe the degree of association between genes.

Equation 3: TOM=(∑_μ≠ij_α_iμ_α_μj_+α_ij_)/(min(∑_μ_α_iμ_+∑_μ_α_jμ_)+1-α_ij_)
TOM indicates the degree of dissimilarity between gene i and gene j. Hierarchical clustering of genes using 1-TOM as a distance, and then using the method of dynamic cut tree for module identification. The most representative gene in each module is called the eigenvector gene, referred to as ME, which represents the overall level of gene expression within the module. It is the first principal component in each module, and Equation 4 is used to calculate the ME. Where i represents the gene in module q and i represents the chip sample in module q.

Equation 4: ME=princomp(x_ij_^q^)
We use the Pearson correlation of the expression profile of a gene in all samples with the Pearson correlation of a feature vector gene expression profile to measure the identity of the gene in the module, i.e., module membership, or MM. Calculate MM using Equation 5, where ME^q^ represents the expression profile of the ith gene, representing the eigenvector gene (ME) of module q, and MMiq represents the identity of gene i in module q. When MM_i_^q^=0, then gene i is not present. The closer the module q, MM_i_^q^ is to +1 or −1, the higher the correlation between gene i and module q. The sign indicates whether the gene i is positively or negatively related to the module q.

Equation 5: MM_i_^q^=cor(x_i_,ME^q^)
Gene significance, referred to as GS, is used to measure the degree of association between genes and external information. The higher the GS, the more biologically significant the gene is, GS=0, indicating that the gene does not participate in the biological problem under study.
