# Supplementary figures and images for: Gene co-expression network for analysis of plasma exosomal miRNAs in the elderly as markers of aging and cognitive decline
Source: PeerJ. 2020 Jan 6;8:e8318. doi: 10.7717/peerj.8318 (PMC6951281; doi:10.7717/peerj.8318)

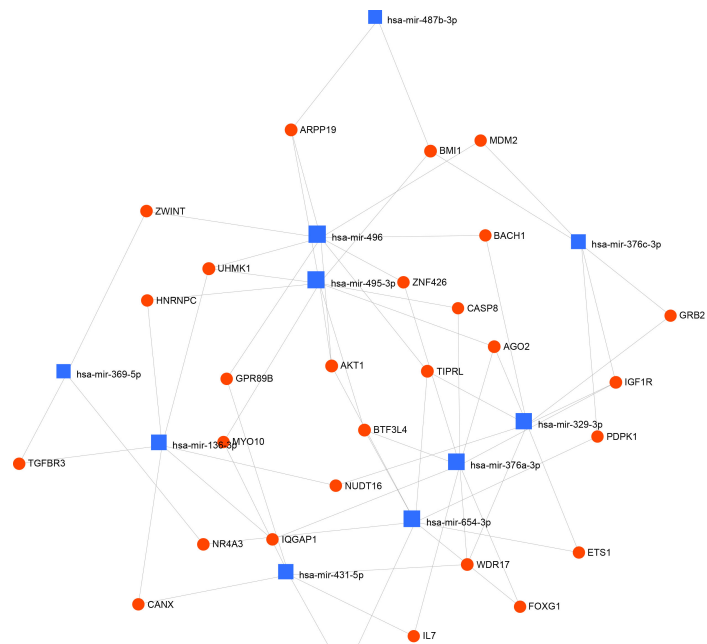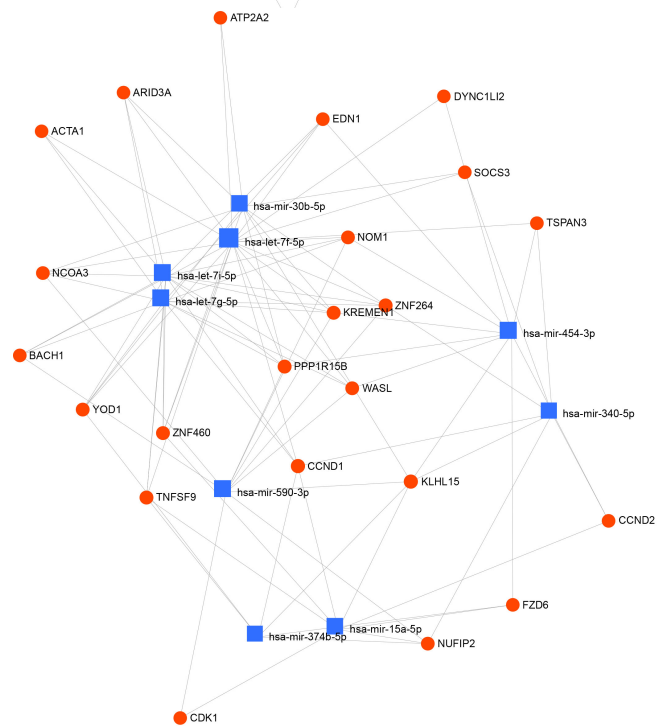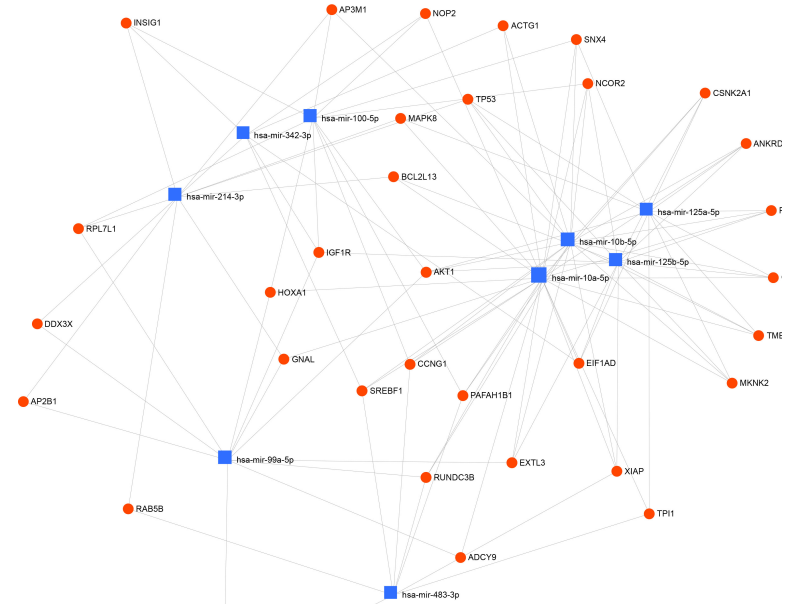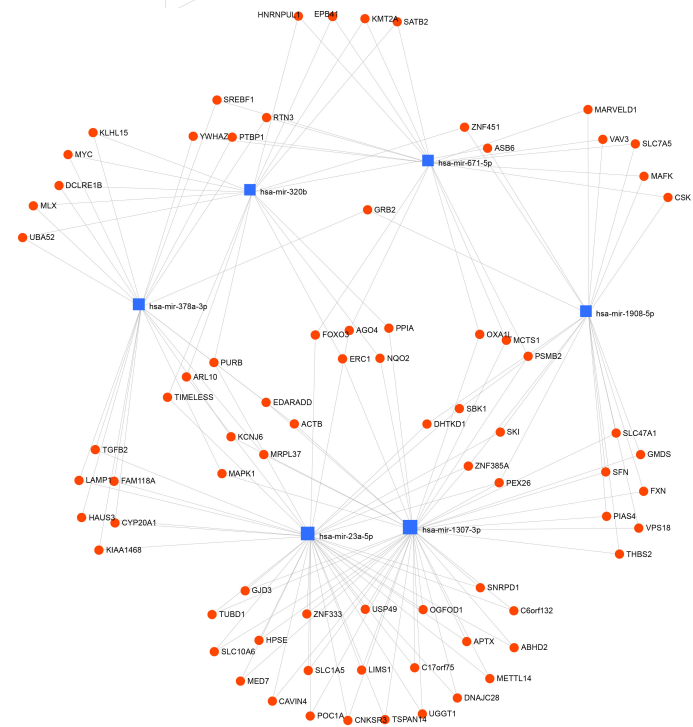

Supplement: Supplemental Information 2 — We subtracted the target gene with the node equal to 1 from the network map and obtained the cut-down network map. Each Figure (A–D) corresponds to the subgraph in Fig. 7. [file peerj-08-8318-s002.pdf]
