# Supplementary material for: Gene co-expression network for analysis of plasma exosomal miRNAs in the elderly as markers of aging and cognitive decline
Source: PeerJ. 2020 Jan 6;8:e8318. doi: 10.7717/peerj.8318 (PMC6951281; doi:10.7717/peerj.8318)
Supplement: Supplemental Information 4 [file peerj-08-8318-s004.pdf]

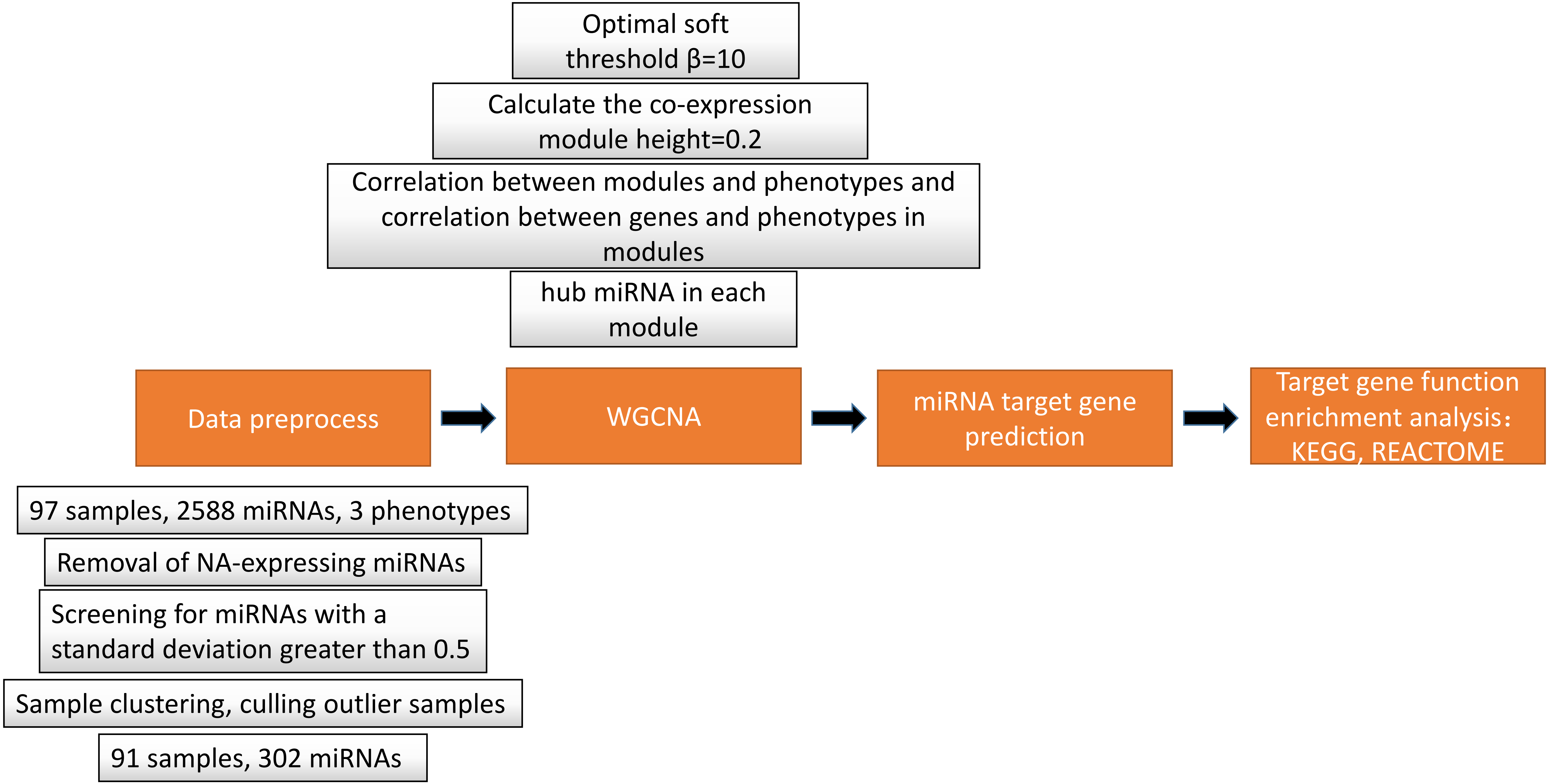
Optimal soft threshold β=10
Calculate the co-expression module height=0.2
Correlation between modules and phenotypes and correlation between genes and phenotypes in modules
hub miRNA in each module
Data preprocess
WGCNA
miRNA target gene prediction
Target gene function enrichment analysis：KEGG, REACTOME
97 samples, 2588 miRNAs, 3 phenotypes
Removal of NA-expressing miRNAs
Screening for miRNAs with a standard deviation greater than 0.5
Sample clustering, culling outlier samples
91 samples, 302 miRNAs
